# Effects of a smartphone mobile application on quality of bowel preparation: A randomized controlled trial

**DOI:** 10.12669/pjms.41.7.11852

**Published:** 2025-07

**Authors:** Selim Demirci, Semih Sezer

**Affiliations:** 1 Selim Demirci Department of Gastroenterology, Dr. Abdurrahman Yurtaslan Oncology, Training and Research Hospital, Ankara, Turkey; 2 Semih Sezer Department of Gastroenterology, Dr. Abdurrahman Yurtaslan Oncology, Training and Research Hospital, Ankara, Turkey

**Keywords:** Bowel preparation, Colonoscopy, Mobile applications, Smartphone

## Abstract

**Objective:**

Successful colonoscopy depends on adequate bowel preparation, while inadequate preparation increases the risk of missed lesions, procedural difficulties, longer durations, and higher costs. This study evaluated the effectiveness of a smartphone application (KOLONAPP) delivering personalized bowel preparation instructions compared to standard written guidelines in improving preparation quality and patient experience.

**Methods:**

A randomized controlled trial was performed at the gastroenterology endoscopy unit of Abdurrahman Yurtaslan Oncology Hospital, Turkey, between June to August 2024. Patients scheduled for outpatient colonoscopy were randomly assigned to either the app group or control group. The control group received standard written preparation instructions, while the app group used KOLONAPP, featuring visuals, a dietary plan, laxative instructions, recommendations, and reminders. Both groups used laxatives containing Senna A + B. The Boston Bowel Preparation Scale (BBPS) was used to assess bowel preparation quality.

**Results:**

A total of 104 patients underwent colonoscopy, with 52 in each group. The app group achieved significantly higher mean BBPS scores (7.27 ± 1.52) compared to the control group (6.15 ± 1.37; P <0.05). Adjusted analyses revealed that poor bowel preparation was four times more likely in the control group than the app group (odds ratio 4.274, 95% CI, 1.041–17.440; P = 0.044). The app group also showed better dietary adherence and reported a more positive procedural experience.

**Conclusions:**

Personalized smartphone applications like KOLONAPP can significantly improve bowel preparation quality, enhance dietary compliance, and create a better overall patient experience during colonoscopy preparation.

*Clinical Trials Registration No. NCT06569277.

## INTRODUCTION

Colonoscopy, a specific type of endoscopic procedure, is commonly utilized for diagnosing and managing disorders of the lower gastrointestinal tract.^1^ Colonoscopy enables physicians to identify lesions, collect biopsy samples, and perform interventions.^2^,^3^ Thorough bowel preparation is fundamental for colonoscopy, ensuring the best possible visualization of the colon, which in turn enhances the detection of colorectal cancers and minimizes the risk of missing lesions such as polyps. Early detection of polyps through screening is essential before they progress to cancer within the ten years development period.^4^ Also, poor bowel preparation may result in longer colonoscopy procedures, more frequent repeat examinations, and an increased risk of colonoscopy-related complications.^5^

Thorough patient education and consistent guidance are essential for enhancing adherence to bowel preparation protocols.^6^ To improve patients’ comprehension of bowel preparation and ensure adherence to diet, various educational approaches have been utilized, such as illustrations, informative booklets, phone calls, SMS reminders, and smartphone apps.^7^-^9^ However, phone calls and SMS messages require staff effort and technical resources, whereas illustrations and booklets incur additional costs. Smartphone applications for education and follow-up may offer benefits, such as easy portability and extensive accessibility. This study sought to evaluate the effects of personalized bowel preparation instructions delivered through a smartphone application (KOLONAPP) compared to standard written guidelines to enhance the quality of bowel preparation and assess patient experience during the preparation process.

## METHODS

A randomized controlled trial was conducted at the endoscopy unit of gastroenterology clinics at the Abdurrahman Yurtaslan Oncology Training and Research Hospital in Turkey’s capital from June to August 2024. Patients were divided into two groups: smartphone mobile application (app) group and control group. Colonoscopy physicians and nurses were blinded to patient group allocation. The ClinicalTrials identifier for this study is NCT06569277.

### Ethical approval:

It was obtained from the hospital’s clinical ethics committee before the study began (No. 2024-02/41). All the patients provided verbal and written informed consent.

### Inclusion & Exclusion Criteria:

Outpatients aged ≥ 18 years who underwent elective colonoscopies were included in this study. Patients with a history of colonoscopy, those taking anticholinergic or gastrointestinal motility-reducing medications, those with severe chronic kidney disease, heart failure, or a history of abdominal surgery, and those without an Android-based smartphone were excluded from the study.

Demographic and clinical data were collected at enrollment. Patients were randomly assigned to the app or control group using a block randomization method (block size: two). An independent researcher determined the block size. Colonoscopy appointments were scheduled on specific days to streamline data collection. Patients in the app group downloaded a free Android-compatible application, which activated a reminder five days before the procedure. The endoscopy unit’s patient education nurse emphasized the importance of strictly adhering to the recommendations of the application and dietary guidelines for effective bowel preparation. The control group received written instructions on diet and laxative use.

### Bowel Preparation Procedure:

Our institution follows a standardized bowel preparation protocol, including a low-residue diet starting five days before colonoscopy. Patients consumed two X-M 250 ml solutions (750 mg sennoside A+B calcium) mixed with two liters of water at 6 PM the day before the procedure. On the procedure day, two BT enemas (210 mL) were administered, completing bowel cleansing.

### Mobile application (KOLONAPP):

**Fig.1:**
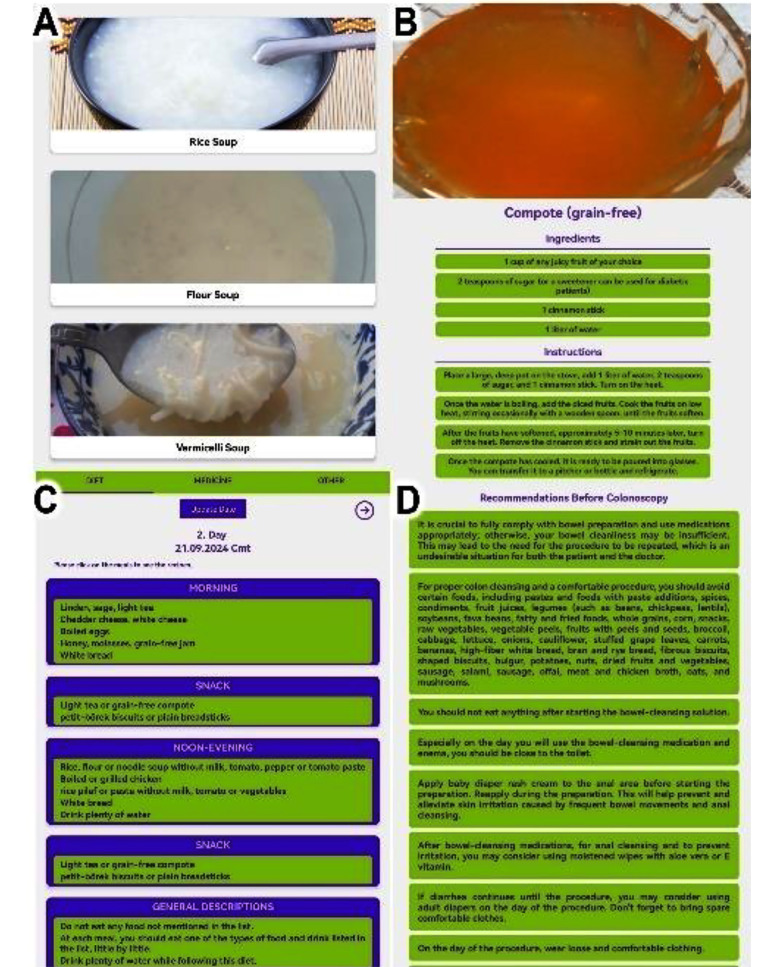
Sections from the application. (A) Soup recipes for a low-residue diet. (B) Step-by-step compote recipe. (C) Dietary plan for the second day. (D) Recommendations before colonoscopy.

The printed brochure designed for colonoscopy bowel preparation included detailed instructions for using bowel preparation medications and a comprehensive 5-day dietary plan, which lists permissible foods and meal suggestions. Patients in the app group downloaded KOLONAPP via QR codes, a free Android application developed using React Native by a gastroenterologist and an IT engineer. The app mirrored the printed diet plan but included images and step-by-step recipes. Users entered their colonoscopy date, triggering reminders five days prior. The “Pre-Colonoscopy Recommendations” tab provided written versions of verbal instructions. Both groups received one-on-one nursing guidance, with instructions reiterated until fully understood. KOLONAPP supports English and Turkish (Fig.1).

### Outcomes:

Bowel preparation quality was assessed using the Boston Bowel Preparation Scale (BBPS), a validated tool rating bowel cleansing in three colon segments (left, transverse, right) on a 0–9 scale, with ≥6 considered adequate.^10^ Secondary outcomes included polyp detection, cecal insertion time, withdrawal time, and patient experience. Two experienced endoscopists (>10 years) performed all elective colonoscopies using a Fujinon EC-590WL4colonoscope without sedation due to high patient volume. A charge nurse recorded insertion and withdrawal times, while all polyps were removed during withdrawal. Patient experience was rated on a 3-point scale (“better than expected,” “as expected,” “worse than expected”). Compliance with dietary and laxative instructions was documented.

### Statistical Analysis and Sample Size:

In a previous study used to calculate the sample size, the mean BBPS score was found to be 7.2 in the app group and 5.9 in the control group.^11^ Based on the assumption that a 1.3-point difference would be considered significant, with an effect size of d=0.74, 95% power, and a significance level of 0.05, 98 patients (≥49 per group) were required for the study. SPSS version 27 was used for statistical analysis. The mean ± standard deviation was used for continuous variables, and normality checks were performed for each variable. For categorical data, the Chi-square test was used, whereas continuous data were analyzed using either the Mann-Whitney U test or Student’s t-test. Logistic regression was applied to identify the factors influencing poor bowel preparation (BBPS score <6 or a segment score <2 in all three segments). Statistical significance was set at P <0.05.

## RESULTS

**Fig.2:**
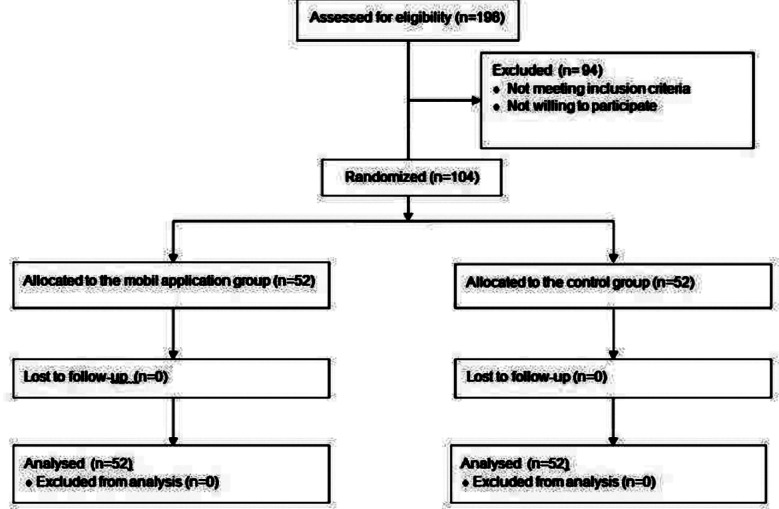
Flowchart of the study.

Between June and August 2024, data were collected from 198 patients, of whom 94 were excluded due to not meeting the inclusion criteria or declining participation. Ultimately, 102 patients completed the colonoscopy. No side effects related to the use of mobile devices were reported. Both the mobile app and control groups achieved a 100% cecal intubation rate, with no significant procedural complications (Fig.2).

Patients’ demographic and clinical characteristics are summarized in Table-I, showing no significant differences in age, sex, BMI, education, marital status, colonoscopy indications, comorbidities, or constipation (P>0.05). Dietary compliance was significantly higher in the app group (P<0.05), while polyp detection rates and cecal insertion times were comparable (P>0.05). The app group exhibited a significantly shorter withdrawal time (P<0.05) and reported a more positive colonoscopy experience (57.7% vs. 34.6%).

**Table-I:** Patient Characteristics and Clinical Data.

Characteristics	App group (n=52)	Control (n=52)	P value
Age (year)	37.94 ± 6.67	40.85 ± 10.43	0.094
Gender, male, n (%)	32 (61.5)	28 (53.8)	0.427
BMI (kg/m^2^)	29.76 ± 5.71	28.03 ± 4.34	0.085
Education level, n (%)			0.428
Elementary school	9 (17.3)	15 (28.8)	
Secondary school	9 (17.3)	11 (21.2)	
High school	20 (38.5)	15 (28.8)	
University	14 (26.9)	11 (21.2)	
Marital status, n (%)			0.833
Single	16 (30.8)	17 (32.7)	
Married	36 (69.2)	35 (67.3)	
Colonoscopy indication, n (%)			0.761
Screening	21 (40.4)	23 (44.2)	
Surveillance	12 (23.1)	9 (17.3)	
Symptoms	19 (36.5)	20 (38.5)	
Comorbidities, n (%)			
Diabetes mellitus	13 (25)	17 (32.7)	0.387
Hypertension	11 (21.2)	13 (25)	0.642
Hypothyroidism	6 (11.5)	5 (9.6)	0.750
Constipation, n (%)	12 (23.1)	15 (28.8)	0.502
Compliance with laxative instructions, n (%)			0.791
Yes	43 (82.7)	44 (84.6)	
No	9 (17.3)	8 (15.4)	
Compliance with dietary instructions, n (%)			0.047
Yes	46 (88.5)	38 (73.1)	
No	6 (11.5)	14 (26.9)	
Procedure time (minutes)			
Insertion time	5.92 ± 1.46	6.09 ± 1.48	0.862
Withdrawal time	5.68 ± 1.22	6.43 ± 1.71	0.028
Patients with polyps, n (%)	19 (36.5)	16 (30.8)	0.534
Patient experience			0.036
Better than expected	30 (57.7)	18 (34.6)	
As expected	16 (30.8)	20 (38.5)	
Worse than expected	6 (11.5)	14 (26.9)	

Values are presented as the mean±SD and number (%). Statistically significant values are marked as bold.

BBPS scores for both groups are detailed in Table-II. The app group had a significantly higher mean BBPS score (7.27 ± 1.52) than the control group (6.15 ± 1.37, P<0.001). Bowel cleansing was significantly better in all colon segments in the app group. Additionally, a greater proportion of app users achieved adequate preparation (BBPS ≥ 6, segmental scores ≥ 2 in all segments) compared to the control group (84.6% vs. 63.5%, P = 0.014).

**Table-II:** Cleansing score assessed by BBPS: global score and three-colon segment score.

	App group (n=52)	Control (n=52)	P value
BBPS (≥6)	7.27 ± 1.52	6.15 ± 1.37	<0.001
Left colon	2.63 ± 0.52	2.23 ± 0.58	<0.001
Transvers colon	2.34 ± 0.65	2.01 ± 0.61	0.010
Right colon	2.26 ± 0.71	1.92 ± 0.70	0.015
Good bowel preparation (BBPS ≥6 and three-colon segment scores≥2), no, (%)	44 (84.6)	33 (63.5)	0.014

*Values are presented as the mean ± SD. Statistically significant values are marked as bold. BBPS; Boston Bowel Preparation Scale*

The binary logistic regression analysis for predicting poor bowel preparation (Table-III) revealed that, after adjusting for confounders, the control group had a fourfold higher risk than the app group (OR: 4.274, 95% CI: 1.041–17.550; *P* = 0.044). Additionally, diabetes and non-adherence to laxative or dietary instructions were significant risk factors for inadequate bowel preparation (*P*< 0.05).

**Table-III:** Logistic regression analysis for poor bowel preparations (BBPS<6 orthree-colon segment scores<2).

	Multivariate		
	OR	95 % CI	P value
Age	1.046	0.968 – 1.130	0.258
Sex			
Female	Reference		
Male	1.583	0.476 – 5.261	0.453
BMI	1.040	0.914 – 1.184	0.549
Educational level			
University	Reference		
Primary school	1.287	0.164 – 10.124	0.811
Secondary school	2.140	0.285 – 16.049	0.459
High school	2.277	0.397 – 13.079	0.356
Marital status			
Married	Reference		
Single	2.626	0.726 – 9.501	0.141
Diabetes			
No	Reference		
Yes	4.053	1.067 – 15.397	0.040
Hypertension			
No	Reference		
Yes	1.438	0.327 – 6.320	0.631
Hypothyroidism			
No	Reference		
Yes	3.996	0.642 – 24.862	0.137
Constipation			
No	Reference		
Yes	1.17	0.320 – 4.278	0.812
Compliance with laxative instructions			
Yes	Reference		
No	7.904	1.705 – 36.640	0.008
Compliance with diet instructions			
Yes	Reference		
No	4.902	1.152 – 20.859	0.031
Group			
App group	Reference		
Non-app group	4.274	1.041 – 17.550	0.044

*Statistically significant values are presented in bold.OR ;odds ratio, CI; 95% confidence interval*

## DISCUSSION

Our study is the first randomized controlled trial to evaluate a mobile app that includes step-by-step instructions for low-residue diet recipes in addition to pre-colonoscopy recommendations and laxative use guidelines for colonoscopy preparation. According to the findings of our study, the use of a mobile application that includes meal reminders, colonoscopy instructions, dietary guidelines, meal recipes, and laxative administration guidance has been shown to improve bowel cleansing, enhance adherence to colonoscopy preparation, and enhance the overall patient experience compared to the traditional method.

These results corroborate those of previous studies that demonstrated the beneficial effects of mobile applications in colonoscopy preparation, particularly in improving bowel cleanliness and enhancing patient satisfaction. In a study by Pattarapuntakul et al. involving 119 patients, a mobile application that included routine written instructions and sent push notifications for diet initiation and laxative use one and three days before the procedure showed an excellent bowel cleanliness rate (BBPS ≥8) of 93% in the app group compared to 74.2% in the control group. Similar to our research, the withdrawal time was extended in the control group, but no significant difference in the proportion of patients with polyps was observed between the two groups.^9^

In a study by Lorenzo-Zúñiga et al. involving 280 patients, a mobile app that incorporated visual aids with standard written instructions and sent push notifications for diet initiation was used to assess bowel cleanness using the Harefield Cleansing Scale (HCS). The study found a successful bowel preparation rate of 100% in the app group compared to 96.1% in the control group (P = 0.037). In addition, patients in the intervention group reported a significantly higher level of tolerability and a more positive experience with the prescribed bowel preparation regimen.^12^ In a study conducted by Chenet al., involving 80 patients, a mobile app incorporating visual aids, flowcharts, videos, and push notifications for diet initiation was used to assess bowel preparation using the Aronchick scale. The study found a significantly higher successful bowel preparation rate in the app group (75%) than in the control group (50%) (P = 0.016).^13^ Guo et al. evaluated a mobile app providing education on pre-colonoscopy diet, low-residue diet recommendations, and purgative use. BBPS scores indicated significantly higher bowel preparation success (77.2% vs. 56.8%, P<0.001) and adenoma detection rates (21.4% vs. 12.8%, P = 0.029) in the app group.^14^ Given the similarly positive outcomes observed in our study, the ease, cost-effectiveness, and efficacy of mobile applications in colonoscopy preparation highlight their potential for broader implementation. Further research is warranted to explore their applicability in various patient education domains, expanding their role beyond bowel preparation to other areas of medical adherence and procedural guidance.

Colonic adenomatous polyps can progress from benign to malignant in asymptomatic patients.^15^ Therefore, inadequate bowel preparation is important because of its association with lower polyp detection rates and higher costs. Rex et al. found that inadequate bowel preparation resulted in a 45% reduction in polyp detection and a 5% increase in incomplete or canceled procedures.^16^ In a study conducted by Chokshi et al., repeat colonoscopies were performed on 133 patients with inadequate bowel preparation, detecting at least one adenoma in 33.8% of the patients and identifying high-risk conditions(defined as having three or more adenomas, an adenoma of 1 cm or larger, or the presence of any villous adenoma or high-grade dysplasia) in 18.0%.^17^ While our study showed more polyps in the app group, the difference was not significant. Given the importance of polyp detection, the correlation between bowel cleanliness and polyp detection in our study suggests that mobile applications may enhance polyp detection in future large-scale studies.

In contrast to some studies that identified male sex and pre-existing constipation as risk factors for inadequate bowel preparation, our study did not find male sex or existing constipation as significant risk factors for poor bowel cleanliness.^18^,^19^ This discrepancy may be attributed to the fact that men were reported to have difficulty fully adhering to laxative and enema usage recommendations, and constipated patients already face defecation challenges. In our study, the primary risk factors for inadequate bowel preparation were poor adherence to dietary and laxative recommendations, diabetes, and a lack of mobile app usage. Diabetes has emerged as an independent risk factor for inadequate bowel cleanliness, which aligns with previous studies reporting a higher prevalence of poor bowel preparation in patients with diabetes.^20^

### Strengths

Our study has several strengths, including its design as a randomized controlled trial, in which colonoscopists were blinded to the patient groups, ensuring robust objective evidence. Another notable strength of this study is that, by utilizing a senna-based regimen rather than more potent agents for bowel preparation, the effect of the mobile application on bowel cleanliness, which is the primary outcome of the study, can be assessed more accurately.

### Limitations

The lack of double-blinding is a limitation. The patients’ knowledge of using the mobile app to ensure adequate bowel preparation may have influenced their adherence to bowel preparation recommendations, potentially leading to improved preparation quality. Another limitation may be the patients’ limited access to smartphones and the challenges they face in adapting to digital technologies, which could hinder the effective use of the application. Furthermore, as this study was conducted at a single tertiary center in Turkey, the findings may not be fully generalizable. However, similar benefits may be anticipated in outpatient settings with comparable healthcare structures. Further multicenter studies are needed to validate these results across diverse populations.

## CONCLUSION

Inadequate bowel preparation was mostly due to poor adherence to dietary and laxative instructions, emphasizing the critical importance of patient compliance in successful colonoscopy preparation. In conclusion, KOLONAPP improved bowel preparation and patient experience through personalized guidance, visual aids, and medication reminders while reducing costs. Future artificial intelligence driven advancements may enable interactive programs to optimize dietary plans, fluid intake, and bowel monitoring, allowing automated rescheduling and personalized adjustments.
